# A Dunnett-Type Test and Its Sample Size Calculation for Comparing *K* ROC Curves with a Control

**DOI:** 10.3390/diagnostics14161813

**Published:** 2024-08-20

**Authors:** Sin-Ho Jung

**Affiliations:** Department of Biostatistics and Bioinformatics, Duke University, Durham, NC 27705, USA; sinho.jung@duke.edu

**Keywords:** AUC, biomarker, family-wise error rate, location-shift model, prevalence, sensitivity, specificity

## Abstract

Diagnostic biomarkers are key components of diagnostics. In this paper, we consider diagnostic biomarkers taking continuous values that are associated with a dichotomous disease status, called malignant or benign. The performance of such a biomarker is evaluated by the area under the curve (AUC) of its receiver operating characteristic curve. We assume that, together with the disease status, one control and multiple experimental biomarkers are collected from each subject to test if any of the experimental biomarkers have a larger AUC than the control. In this case, each experimental biomarker will be compared with the control so that a multiple testing issue is involved in the comparisons. In this paper, we propose a simple non-parametric statistical testing procedure to compare K(≥2) experimental biomarkers with a control, adjusting for the multiplicity and its sample size calculation method. Our sample size formula requires the specification of the AUC values (or the standardized effect size of each biomarker between the benign and malignant groups) together with the correlation coefficients between the biomarkers, the prevalence of the malignant group in the study population, the type I error rate, and the power. Through simulations, we show that the statistical test controls the overall type I error rate accurately and the proposed sample size closely maintains the specified statistical power.

## 1. Introduction

Different types of biomarkers are measured from the tumor, blood, or urine using molecular, biochemical, physiological, anatomical, or imaging methods. The observed biomarkers are used for various purposes during the diagnosis and treatment of diseases. For example, diagnostic biomarkers are used to diagnose diseases, predictive biomarkers are used to predict the response to specific treatments, and prognostic biomarkers are used to measure the aggressiveness of a disease for patients with no treatment or a non-targeted treatment. When the outcome is dichotomous and the biomarker is continuous, the performance of a biomarker is evaluated by the area under the curve (AUC) of its receiver operating characteristic (ROC) curve, which plots the sensitivity against one minus the specificity calculated for all the possible cutoff values of the biomarker. In this paper, we consider diagnostic biomarkers taking continuous values that are associated with a dichotomous disease status, called benignity or malignancy, but the proposed methods can be used for any type of biomarker with such properties.

Various parameters have been used to measure the performance of biomarkers and compare among different biomarkers. For instance, sensitivity at a fixed specificity level determined by the cutoff value of each biomarker may be compared between biomarkers [1,2]. Some investigators proposed testing methods to compare the whole or partial AUC of ROC curves of biomarkers [3,4,5,6,7].

Emir et al. [8] extended the method of Delong et al. [6] to comparison of ROC curves from longitudinally collected biomarkers, and Jung et al. [9] proposed an ROC-type method to compare the performance of biomarkers that are associated with a time-to-event endpoint such as progression-free survival.

Sample size calculation is an important step in the development stage of medical projects. Hwang and Su [10] proposed a sample size formula for the test statistic of Delong et al. [6]. Their variance formula requires the specification of so many parameter values. Furthermore, these parameters are not conceptually interpretable, so it is almost impossible to accurately specify these parameters in sample size calculations. This can prevent us from using their sample size formula in designing real biomarker projects. Jung [11] proposed a simple non-parametric test statistic that is asymptotically identical to that of Delong et al. [6] and its sample size formula. Compared to the formula of Hwang and Su [10], his formula requires specification of much smaller number of design parameters and the design parameters are intuitively interpretable. These sample size methods are limited to the comparison of two biomarkers.

In this paper, we consider the cases where a control biomarker (usually an existing one) and K(≥2) experimental biomarkers are collected from each subject together with a dichotomous disease status. We want to discover the experimental biomarkers that are more closely associated with the disease outcome than the control biomarker. To this end, we compare the AUC of the ROC curve of each experimental biomarker with that of the control using the two-sample test statistic [11]. With *K* experimental biomarkers to be compared with the control, we will conduct *K* statistical tests, so the discovery of new biomarkers will involve a multiple testing issue. We propose a multiple testing procedure using a Dunnett-type approach [12,13]. We derive a sample size formula for the multiple testing procedure too. While the statistical test is completely non-parametric, its power depends on the distributions of the biomarkers. Furthermore, the biomarkers to be compared are correlated because they are collected from each patient. This results in additional problems in deriving the statistical testing method and its sample size formula.

Since the AUC of an ROC curve is a rank statistic, it is invariant to the monotone transformations of biomarker values. Hence, if we find a transformation of biomarker distributions to symmetric distributions, such as normal distributions, with a location-shift model and unit variances, then, for a sample size calculation, we need to specify only the location parameter between the benign and malignant groups for each biomarker (which corresponds to the AUC) and the correlation coefficient between the paired biomarkers after the transformation. A log-transformation followed by standardization often carries out this goal. Through simulations, we show that our multiple testing procedure accurately controls the overall type I error rate, called the family-wise error rate (FWER), and its sample size formula closely maintains a specified statistical power.

## 2. A Dunnett-Type Test to Compare *K* Biomarkers with a Control

Suppose that biomarker 0 denotes a control (or existing) biomarker and biomarkers 1,…,K denote *K* experimental biomarkers that are collected from each subject. Let $X_{k}$ and $Y_{k}$ denote the random variables for observations of biomarker *k*(=0, 1, …,K) from malignant and benign cases, respectively. In a data set, we have copies of $X_{k}$, $\left(x_{k1},\ldots ,x_{k,m}\right)$, from *m* malignant cases, and copies of $Y_{k}$, $\left(y_{k1},\ldots ,y_{k,n}\right)$, from *n* benign cases. For the total sample size *N*(=m+n), m/N→γ∈(0,1) denotes the prevalence of malignancy in the target population. As can be seen from our sample size formula and the results of numerical studies in Section 5, our proposed test will have a maximum power if benign and malignant cases are balanced, i.e., γ=1/2. Let $F_{k}\left(t\right)=P\left(X_{k}\leq t\right)$ and $G_{k}\left(t\right)=P\left(Y_{k}\leq t\right)$ denote the cumulative density function (CDF) of biomarker *k* for malignant cases and benign cases, respectively, and ${\hat{F}}_{k}\left(t\right)=m^{-1}\sum_{i=1}^{m}I\left(x_{ki}\leq t\right)$ and ${\hat{G}}_{k}\left(t\right)=n^{-1}\sum_{j=1}^{n}I\left(y_{kj}\leq t\right)$ be their empirical estimators.

Without loss of generality, we assume that the raw biomarker values are non-negative and a large biomarker value is associated with a higher chance of malignancy. As such, for a cutoff values *t*, ${\bar{F}}_{k}\left(t\right)=1-F_{k}\left(t\right)$ is called the sensitivity and $G_{k}\left(t\right)$ is called the specificity of biomarker *k*. For ${\bar{G}}_{k}\left(t\right)=1-G_{k}\left(t\right)$, the ROC curve of biomarker *k* is the plot of $\left({\bar{G}}_{k}\left(t\right),{\bar{F}}_{k}\left(t\right)\right)$ with respect to all possible cutoff value *t*.

AUC of the ROC curve for biomarker *k* is $\theta_{k}=P\left(X_{k}\geq Y_{k}\right)=\int_{0}^{\infty }G_{k}\left(t\right)dF_{k}\left(t\right)$, which is estimated by

$${\hat{\theta }}_{k}=\int_{0}^{\infty }{\hat{G}}_{k}\left(t\right)d{\hat{F}}_{k}\left(t\right)=\frac{1}{mn}\sum_{i=1}^{m}\sum_{j=1}^{n}I\left(x_{ki}\geq y_{kj}\right)$$

For experimental biomarker k(=1,…,K), we want to test the null hypothesis $H_{k}:\theta_{k}=\theta_{0}$ against an alternative hypothesis ${\bar{H}}_{k}:\theta_{k}>\theta_{0}$ using ${\hat{\theta }}_{k}-{\hat{\theta }}_{0}$. It was shown that [11], for k=0,1,…,K, we have

(1) $${\hat{\theta }}_{k}-\theta_{k}=m^{-1}\sum_{i=1}^{m}\epsilon_{ki}+n^{-1}\sum_{j=1}^{n}e_{kj}+o_{p}\left(N^{-1/2}\right)$$

where $\epsilon_{ki}=\int_{0}^{\infty }G_{k}\left(t\right)d\left\{I\left(x_{ki}\leq t\right)-F_{k}\left(t\right)\right\}=G_{k}\left(x_{ki}\right)-\theta_{k}$ and $e_{kj}=\int_{0}^{\infty }\left\{I\left(y_{kj}\leq t\right)-G_{k}\left(t\right)\right\}dF_{k}\left(t\right)={\bar{F}}_{k}\left(y_{kj}\right)-\theta_{k}$. Hence, for k=1,…,K,

(2) $$\sqrt{N}\left({\hat{\theta }}_{k}-{\hat{\theta }}_{0}\right)=\sqrt{N}\left\{m^{-1}\sum_{i=1}^{m}\left(\epsilon_{ki}-\epsilon_{0i}\right)+n^{-1}\sum_{j=1}^{n}\left(e_{kj}-e_{0j}\right)\right\}+\Delta_{k}\sqrt{N}+o_{p}\left(1\right)$$

where $\Delta_{k}=\theta_{k}-\theta_{0}$.

Note that $\left(\epsilon_{0i},\epsilon_{1i},\ldots ,\epsilon_{Ki}\right)$ and $\left(e_{0i},e_{1i},\ldots ,e_{Ki}\right)$ are K+1-dimensional independent random vectors with mean 0 because malignant and benign groups are independent. Furthermore, under $H_{k}$, we have $\Delta_{k}=0$, so that, by the central limit theorem (CLT), $\sqrt{N}\left({\hat{\theta }}_{k}-{\hat{\theta }}_{0}\right)/{\hat{\sigma }}_{k}$ converges to N(0,1), where

$${\hat{\sigma }}_{k}^{2}=N\left\{m^{-2}\sum_{i=1}^{m}{\left({\hat{\epsilon }}_{ki}-{\hat{\epsilon }}_{0i}\right)}^{2}+n^{-2}\sum_{j=1}^{n}{\left({\hat{e}}_{kj}-{\hat{e}}_{0j}\right)}^{2}\right\}$$

and ${\hat{\epsilon }}_{ki}$ and ${\hat{e}}_{kj}$ are obtained from $\epsilon_{ki}$ and $e_{kj}$, respectively, by replacing $F_{k}$ and $G_{k}$ with their consistent estimators ${\hat{F}}_{k}$ and ${\hat{G}}_{k}$. That is,

$${\hat{\epsilon }}_{ki}={\hat{G}}_{k}\left(x_{ki}\right)-{\hat{\theta }}_{k}=\frac{1}{n}\sum_{j=1}^{n}I\left(y_{kj}\leq x_{ki}\right)-{\hat{\theta }}_{k}$$

and

$${\hat{e}}_{kj}=\hat{\bar{F}}\left(y_{kj}\right)-{\hat{\theta }}_{k}=\frac{1}{m}\sum_{i=1}^{m}I\left(x_{ki}\geq y_{kj}\right)-{\hat{\theta }}_{k}$$

The univariate (or unadjusted) test rejects $H_{k}$ if $Z_{k}=\sqrt{N}\left({\hat{\theta }}_{k}-{\hat{\theta }}_{0}\right)/{\hat{\sigma }}_{k}>z_{1-\alpha }$, where $z_{1-\alpha }$ denotes the 100(1−α) percentile of N(0,1) distribution. Appendix A of Jung [11] shows that the variance estimator ${\hat{\sigma }}_{k}^{2}$ is asymptotically identical to that of DeLong et al. [6]. In this sense, our univariate test statistic is asymptotically identical to that of DeLong et al. [6].

When there are multiple experimental biomarkers (i.e., K≥2), the biomarker discovery will have increased false positivity by the multiple univariate tests. For accurate control of false positivity, we need a multiple testing adjustment. We propose to control the FWER, i.e., the probability to accept an experimental biomarker when all *K* values of them are no better than the control. For the overall null hypothesis $H_{0}=\cap_{k=1}^{K}H_{k}$ and a common critical value $c=c_{\alpha }$, controlling the FWER at α is obtained by solving

(3) $$\alpha =P\left\{\cup_{k=1}^{K}\left(Z_{k}>c\right)|H_{0}\right\}$$

with respect to *c*. Note that the critical region for our testing has a square shape. Delong et al. [6] proposed a one-way ANOVA-type test with an ellipsoidal-shape critical value, but our Dunnett-type test will be more appropriate when comparing multiple experimental biomarkers with a common control.

In order to calculate the FWER provided in Equation (3), we need the joint distribution of $\left(Z_{1},\ldots ,Z_{K}\right)$ under $H_{0}$. Applying the multivariate CLT to Equation (2), under $H_{0}$, $\left(Z_{1},\ldots ,Z_{K}\right)$ is asymptotically normal with mean 0 and variance Σ that can be consistently estimated by

$$\hat{\Sigma }={\left({\hat{\rho }}_{kk^{\prime }}\right)}_{k,k^{\prime }=1,\ldots ,K}$$

where ${\hat{\rho }}_{kk^{\prime }}={\hat{\sigma }}_{kk^{\prime }}/\sqrt{{\hat{\sigma }}_{k}^{2}{\hat{\sigma }}_{k^{\prime }}^{2}}$ and

$${\hat{\sigma }}_{kk^{\prime }}=N\left\{m^{-2}\sum_{i=1}^{m}\left({\hat{\epsilon }}_{ki}-{\hat{\epsilon }}_{0i}\right)\left({\hat{\epsilon }}_{k^{\prime }i}-{\hat{\epsilon }}_{0i}\right)+n^{-2}\sum_{j=1}^{n}\left({\hat{e}}_{kj}-{\hat{e}}_{0j}\right)\left({\hat{e}}_{k^{\prime }j}-{\hat{e}}_{0j}\right)\right\}$$

For a given critical value *c*, the FWER is obtained by calculating the probability on the right-hand side of Equation (3) based on the multivariate normal distribution using simulations or a numerical integration method. Finally, the critical value $c=c_{\alpha }$ controlling the FWER at α is obtained by solving Equation (3) with respect to *c* and accepting (or discovering) biomarker k(=1,…,K) if $Z_{k}>c_{\alpha }$.

When K=2, the double integration for the probability calculation can be simplified to a single integration. If $\left(Z_{1},Z_{2}\right)$ is a bivariate normal random vector with mean 0, variance 1, and correlation coefficient $\rho_{12}$, then the conditional distribution of $Z_{1}$ given $Z_{2}=z$ is normal with mean $\rho_{12}z$ and variance $\left(1-\rho_{12}^{2}\right)$. Hence, from Equation (3), we have

$$\mathrm{FWER}=P\left\{\left(Z_{1}>c\right)\cup \left(Z_{2}>c\right)\right\}=1-P\left\{\left(Z_{1}\leq c\right)\cap \left(Z_{2}\leq c\right)\right\}$$

so the critical value $c=c_{\alpha }$ controlling the FWER at α is obtained by solving the equation

(4) $$\alpha =1-\int_{-\infty }^{c}\phi \left(z\right)\Phi \left(\frac{c-{\hat{\rho }}_{12}z}{\sqrt{1-{\hat{\rho }}_{12}^{2}}}\right)dz$$

with respect to *c*, where ϕ(·) and Φ(·) are the probability density function (PDF) and CDF of N(0,1) distribution, respectively.

For biomarker k(=1,…,K) with test statistic value $Z_{k}=z_{k}$, we may calculate the FWER-adjusted *p*-value by

$$p_{k}=P\left(Z_{1}>z_{k},\mathrm{or}\ldots ,\mathrm{or}Z_{K}>z_{k}|H_{0}\right)=P\left\{\cup_{k^{\prime }=1}^{K}\left(Z_{k^{\prime }}>z_{k}\right)|H_{0}\right\}$$

## 3. Sample Size Calculation for the Dunnett-Type Test

Now, we derive a sample size formula for a specified alternative hypothesis $H_{a}=\cup_{k=1}^{K}{\bar{H}}_{k}$. For $\Delta_{k}>0$, we consider a specific alternative hypothesis

$$H_{a}:\theta_{k}-\theta_{0}=\Delta_{k}\;\mathrm{for}\;k=1,\ldots ,K$$

From Equation (2), we have $E\left({\hat{\theta }}_{k}-{\hat{\theta }}_{0}\right)=\Delta_{k}$ for k=1,…,K under $H_{a}$.

We have to calculate the variances and covariances of $\sqrt{N}\left({\hat{\theta }}_{0},{\hat{\theta }}_{1},\ldots ,{\hat{\theta }}_{K}\right)$. Define $\sigma_{k}^{2}\left(\epsilon \right)=\mathrm{var}\left(\epsilon_{ki}\right)$ and $\sigma_{k}^{2}\left(e\right)=\mathrm{var}\left(e_{ki}\right)$, i.e.,

$$\sigma_{k}^{2}\left(\epsilon \right)=\mathrm{var}\left\{G_{k}\left(X_{k}\right)\right\}=\int_{0}^{\infty }G_{k}{\left(x\right)}^{2}dF_{k}\left(x\right)-\theta_{k}^{2}$$

and

$$\sigma_{k}^{2}\left(e\right)=\mathrm{var}\left\{{\bar{F}}_{k}\left(Y_{k}\right)\right\}=\int_{0}^{\infty }{\bar{F}}_{k}{\left(y\right)}^{2}dG_{k}\left(y\right)-\theta_{k}^{2}$$

For $0\leq k\neq k^{\prime }\leq K$, let $f_{kk^{\prime }}\left(x_{k},x_{k^{\prime }}\right)$ and $g_{kk^{\prime }}\left(y_{k},y_{k^{\prime }}\right)$ denote the PDFs of $\left(X_{k},X_{k^{\prime }}\right)$ and $\left(Y_{k},Y_{k^{\prime }}\right)$, respectively. We also define $\sigma_{kk^{\prime }}\left(\epsilon \right)=\mathrm{cov}\left(\epsilon_{ki},\epsilon_{k^{\prime }i}\right)$ and $\sigma_{kk^{\prime }}\left(e\right)=\mathrm{cov}\left(e_{ki},e_{k^{\prime }i}\right)$, i.e.,

$$\sigma_{kk^{\prime }}\left(\epsilon \right)=\mathrm{cov}\left\{G_{k}\left(X_{k}\right),G_{k^{\prime }}\left(X_{k^{\prime }}\right)\right\}=\int_{0}^{\infty }\int_{0}^{\infty }G_{k}\left(x_{k}\right)G_{k^{\prime }}\left(x_{k^{\prime }}\right)f_{kk^{\prime }}\left(x_{k},x_{k^{\prime }}\right)dx_{k}dx_{k^{\prime }}-\theta_{k}\theta_{k^{\prime }}$$

and

$$\sigma_{kk^{\prime }}\left(e\right)=\mathrm{cov}\left\{{\bar{F}}_{k}\left(Y_{k}\right),{\bar{F}}_{k^{\prime }}\left(Y_{k^{\prime }}\right)\right\}=\int_{0}^{\infty }\int_{0}^{\infty }{\bar{F}}_{k}\left(y_{k}\right){\bar{F}}_{k^{\prime }}\left(y_{k^{\prime }}\right)g_{kk^{\prime }}\left(y_{1},y_{2}\right)dy_{k}dy_{k^{\prime }}-\theta_{k}\theta_{k^{\prime }}$$

Let $s_{k}^{2}=\mathrm{var}\left({\hat{\theta }}_{k}\sqrt{N}\right)$ and $s_{kk^{\prime }}=\mathrm{cov}\left({\hat{\theta }}_{k}\sqrt{N},{\hat{\theta }}_{k^{\prime }}\sqrt{N}\right)$ for $0\leq k,k^{\prime }\leq K$. Then, from Equation (1), we have

$$s_{k}^{2}=\gamma^{-1}\sigma_{k}^{2}\left(\epsilon \right)+{\bar{\gamma }}^{-1}\sigma_{k}^{2}\left(e\right)$$

and

$$s_{kk^{\prime }}=\gamma^{-1}\sigma_{kk^{\prime }}\left(\epsilon \right)+{\bar{\gamma }}^{-1}\sigma_{kk^{\prime }}\left(e\right)$$

where $\bar{\gamma }=1-\gamma$.

Hence, we have $V=\mathrm{var}\left\{\sqrt{N}\left({\hat{\theta }}_{1}-{\hat{\theta }}_{0}\right),\ldots ,\sqrt{N}\left({\hat{\theta }}_{K}-{\hat{\theta }}_{0}\right)\right\}={\left(\sigma_{kk^{\prime }}\right)}_{k,k^{\prime }=1,\ldots ,K}$ with

$$\sigma_{kk}=\sigma_{k}^{2}=\mathrm{var}\left\{\sqrt{N}\left({\hat{\theta }}_{k}-{\hat{\theta }}_{0}\right)\right\}=s_{k}^{2}-2s_{k0}+s_{0}^{2}$$

and, for $k\neq k^{\prime }\left(=1,\ldots ,K\right)$,

$$\sigma_{kk^{\prime }}=\mathrm{cov}\left\{\sqrt{N}\left({\hat{\theta }}_{k}-{\hat{\theta }}_{0}\right),\sqrt{N}\left({\hat{\theta }}_{k^{\prime }}-{\hat{\theta }}_{0}\right)\right\}=s_{kk^{\prime }}-s_{k0}-s_{k^{\prime }0}+s_{0}^{2}$$

By the multivariate CLT applied to (2) under $H_{a}$, $\sqrt{N}\left({\hat{\theta }}_{1}-{\hat{\theta }}_{0},\ldots ,{\hat{\theta }}_{K}-{\hat{\theta }}_{0}\right)$ is asymptotically normal with mean $\sqrt{N}\left(\Delta_{1},\ldots ,\Delta_{K}\right)$ and variance *V*. So, under $H_{a}$, we have

$$Z_{k}=U_{k}+\frac{\Delta_{k}\sqrt{N}}{\sigma_{k}}$$

where

$$U_{k}=\frac{\sqrt{N}\left({\hat{\theta }}_{k}-{\hat{\theta }}_{0}-\Delta_{k}\right)}{\sigma_{k}}$$

Note that $\left(U_{1},\ldots ,U_{K}\right)$ is normal with mean 0 and variance–covariance of $\Sigma ={\left(\rho_{kk^{\prime }}\right)}_{k,k^{\prime }=1,\ldots ,K}$ with $\rho_{kk}=1$ and $\rho_{kk^{\prime }}=\sigma_{kk^{\prime }}/\left(\sigma_{k}\sigma_{k^{\prime }}\right)$ for $1\leq k\neq k^{\prime }\leq K$.

Hence, for given *N*, the power 1−β of the Dunnett-type test controlling the FWER at α is provided as

$$1-\beta =P\{\cup_{k=1}^{K}\left(Z_{k}>c_{\alpha }\right)|H_{a}\}$$

(5) $$=P\left\{\cup_{k=1}^{K}\left(U_{k}>c_{\alpha }-\frac{\Delta_{k}\sqrt{N}}{\sigma_{k}}\right)\right\}$$

where $c=c_{\alpha }$ is obtained by solving

(6) $$\alpha =P\left\{\cup_{k=1}^{K}\left(U_{k}>c\right)\right\}$$

For given power 1−β, the required sample size *N* is obtained by solving Equation (5) with respect to *N*. The probabilities on the right-hand sides of Equations (5) and (6) can be solved using simulations or a multivariate numerical integration.

When K=2, the conditional distribution of $U_{1}$ given $U_{2}=u$ is normal with mean $\rho_{12}u$ and variance $\left(1-\rho_{12}^{2}\right)$, so Equations (5) and (6) are simplified to

(7) $$1-\beta =1-\int_{-\infty }^{{\bar{c}}_{1}}\phi \left(u\right)\Phi \left(\frac{{\bar{c}}_{2}-\rho_{12}u}{\sqrt{1-\rho_{12}^{2}}}\right)du$$

and

(8) $$\alpha =1-\int_{-\infty }^{c_{\alpha }}\phi \left(u\right)\Phi \left(\frac{c_{\alpha }-\rho_{12}u}{\sqrt{1-\rho_{12}^{2}}}\right)du$$

where ${\bar{c}}_{k}=c_{\alpha }-\sigma_{k}^{-1}\Delta_{k}\sqrt{N}$ for k=1,2.

## 4. Transformation of Biomarker Data to Normal Distributions

The Dunnett-type test statistic is completely non-parametric, but the asymptotic distribution and power of the test statistic depend on the distributions of biomarkers for benign and malignant groups through the values of $\theta_{k}$ under $H_{a}$ and the variance–covariance of $\left({\hat{\theta }}_{1}-{\hat{\theta }}_{0},\ldots ,{\hat{\theta }}_{K}-{\hat{\theta }}_{0}\right)$, as shown in the previous section. So, we have to assume a parametric model for sample size calculation.

Biomarkers usually take positive values. If we know the distributions of original biomarker values, then we may calculate a sample size based on them. However, we rarely have good information about the original distributions. Furthermore, for most multivariate positive distributions, their means, variances, and correlation coefficients are mutually associated, so it is difficult to specify the parameter values for a sample size calculation.

Since AUC is a rank statistic, it is invariant to monotone transformations of $X_{k}$ and $Y_{k}$. We can find a transformation, such as logarithm, to change the distribution of a positive random variable to a (approximately) normal distribution, for which the parameters are not associated with each other and can be easily specified. Such a transformation tends to stabilize the variance of random variables too.

We assume that, after a log-transformation, each biomarker marginally has normal distributions with a common variance (less strictly, a location-shift model) between malignant and benign groups. Then, we standardize the data by subtracting the mean of benign group and dividing by the common standard deviation. Then, for the resulting data of biomarker *k*, $X_{k}-\delta_{k}$ and $Y_{k}$ are independent N(0,1) with a location parameter $\delta_{k}\left(>0\right)$. Note that $\delta_{k}$ is the standardized effect size of log-transformed biomarker values between malignant and benign groups. It is known that $\theta_{k}$ and $\delta_{k}$ have a 1-to-1 relationship, i.e., $\theta_{k}=\int_{-\infty }^{\infty }\phi \left(z-\delta_{k}\right)\Phi \left(z\right)dz$ [11].

With such a transformation, it is easy to conceptualize the design parameters for sample size calculations. Let $\phi_{r}\left(x,y\right)$ denote the PDF of bivariate normal distribution with 0 means, unit variances, and correlation coefficient *r*. Then, we have $F_{k}\left(t+\delta_{k}\right)=G_{k}\left(t\right)=\Phi \left(t\right)$, $f_{kk^{\prime }}\left(x_{k},x_{k^{\prime }}\right)=\phi_{r_{kk^{\prime }}}\left(x_{k}-\delta_{k},x_{k^{\prime }}-\delta_{k^{\prime }}\right)$, and $g_{kk^{\prime }}\left(y_{k},y_{k^{\prime }}\right)=\phi_{r_{kk^{\prime }}}\left(y_{k},y_{k^{\prime }}\right)$, where $r_{kk^{\prime }}=\mathrm{corr}\left(X_{k},X_{k^{\prime }}\right)=\mathrm{corr}\left(Y_{k},Y_{k^{\prime }}\right)$. Furthermore, after such a transformation by Appendix B of Jung [11], we have $\sigma_{k}^{2}\left(\epsilon \right)=\sigma_{k}^{2}\left(e\right)$ and $\sigma_{kk^{\prime }}\left(\epsilon \right)=\sigma_{kk^{\prime }}\left(e\right)$, so we have $s_{k}^{2}={\left(\gamma \bar{\gamma }\right)}^{-1}\sigma_{k}^{2}\left(\epsilon \right)$ and $s_{kk^{\prime }}={\left(\gamma \bar{\gamma }\right)}^{-1}\sigma_{kk^{\prime }}\left(\epsilon \right)$, and $\sigma_{k}^{2}$ and $\sigma_{kk^{\prime }}$ are simplified to

(9) $$\sigma_{k}^{2}={\left(\gamma \bar{\gamma }\right)}^{-1}\left\{\sigma_{k}^{2}\left(\epsilon \right)-2\sigma_{k0}\left(\epsilon \right)+\sigma_{0}^{2}\left(\epsilon \right)\right\}$$

and

(10) $$\sigma_{kk^{\prime }}={\left(\gamma \bar{\gamma }\right)}^{-1}\left\{\sigma_{kk^{\prime }}\left(\epsilon \right)-\sigma_{k0}\left(\epsilon \right)-\sigma_{k^{\prime }0}\left(\epsilon \right)+\sigma_{0}^{2}\left(\epsilon \right)\right\}$$

where

$$\sigma_{k}^{2}\left(\epsilon \right)=\int_{-\infty }^{\infty }\Phi^{2}\left(x\right)\phi \left(x-\delta_{k}\right)dx-\theta_{k}^{2}$$

and

$$\sigma_{kk^{\prime }}\left(\epsilon \right)=\int_{-\infty }^{\infty }\int_{-\infty }^{\infty }\Phi \left(x_{k}\right)\Phi \left(x_{k^{\prime }}\right)\phi_{\rho_{kk^{\prime }}}\left(x_{k}-\delta_{k},x_{k^{\prime }}-\delta_{k^{\prime }}\right)dx_{k}dx_{k^{\prime }}-\theta_{k}\theta_{k^{\prime }}$$

Note that the ranges of integrations are changed from (0,∞) for the original data to (−∞,∞) for the transformed data.

From Equations (9) and (10), we find that the sample size depends on the prevalence of malignancy γ through $\gamma \bar{\gamma }$, so the required sample size is minimized when $\gamma =\bar{\gamma }=1/2$ and is unchanged between two γ values with identical |γ−1/2| value. Furthermore, since $\rho_{kk^{\prime }}=\sigma_{kk^{\prime }}/\left(\sigma_{k}\sigma_{k^{\prime }}\right)$, the correlation matrix of $\sqrt{N}\left({\hat{\theta }}_{1}-{\hat{\theta }}_{0},\ldots ,{\hat{\theta }}_{K}-{\hat{\theta }}_{0}\right)$, $\Sigma ={\left(\rho_{k,k^{\prime }}\right)}_{k,k^{\prime }=1,\ldots ,K}$, is free of γ.

Assuming that, for biomarker *k*, $X_{k}$ and $Y_{k}$ marginally follow normal distributions after a log-transformation, a sample size calculation is conducted as follows.

1. Specify
  (a) FWER=α and power=1−β;
  (b) Prevalence of malignancy =γ;
  (c) For biomarker k(=0,1,…,K), standardized effect size $\delta_{k}$ between benign and malignant groups or AUC $\theta_{k}=\int_{-\infty }^{\infty }\phi \left(z-\delta_{k}\right)\Phi \left(z\right)dz$ under $H_{a}$; and
  (d) $r_{kk^{\prime }}=\mathrm{corr}\left(X_{k},X_{k^{\prime }}\right)=\mathrm{corr}\left(Y_{k},Y_{k^{\prime }}\right)$.
2. Calculate $\rho_{kk^{\prime }}=\mathrm{corr}\left({\hat{\theta }}_{k}-{\hat{\theta }}_{0},{\hat{\theta }}_{k^{\prime }}-{\hat{\theta }}_{0}\right)$ using Equations (9) and (10) based on specified $\delta_{k}$ (or $\theta_{k}$) and $r_{kk^{\prime }}$.
3. Calculate $c_{\alpha }$ by solving Equation (6).
4. Calculate the required sample size *N* by solving Equation (5).

## 5. Numerical Studies

Suppose that there are two experimental biomarkers and a control biomarker following the trivariate normal distribution model with a location-shift as discussed in the previous section. We consider prevalence of malignancy γ=0.1, 0.3, or 0.5, $\theta_{0}=0.6$, 0.7, or 0.8, AUCs for biomarkers $\theta_{1}=\theta_{2}=\theta_{0}$ under $H_{0}$, and $\theta_{1}=\theta_{2}=\theta_{0}+\Delta$ with Δ=0.1 or 0.15 under $H_{a}$. Using the one-to-one relationship between AUC $\theta_{k}$ and location-shift parameter $\delta_{k}$ between $X_{k}$ and $Y_{k}$, we identify the location parameters $\left(\delta_{0},\delta_{1},\delta_{2}\right)$ corresponding to the specified $\left(\theta_{0},\theta_{1},\theta_{2}\right)$ values. We assume that correlation coefficients between any pair of biomarkers are identically r=0.2 or 0.4. Under each design setting of $\left(\theta_{0},\Delta ,r,\gamma \right)$, we calculate the critical value $c_{\alpha }$ for 1-sided FWER α=0.05 from Equation (8) and calculate sample size *N* for power 1−β=0.8, 0.85, or 0.9.

We conduct simulations to check if the calculated sample size is accurately powered or not. At first, we generate B=5000 samples of size *N* under the design setting of the sample size calculation. That is, for m=γN and n=(1−γ)N, $\left\{\left(x_{0i},x_{1i},x_{2i}\right),i=1,\ldots ,m\right\}$ and $\left\{\left(y_{0j},y_{1j},y_{2j}\right),j=1,\ldots ,n\right\}$ are independent trivariate normal random vectors with mean vectors $\left(\delta_{0},\delta_{1},\delta_{1}\right)$ and (0,0,0), respectively, but with variances 1 and compound symmetry structure with correlation coefficient *r*. Here, $\delta_{0}$ and $\delta_{1}$ are location parameters corresponding to AUCs $\theta_{0}$ and $\theta_{1}=\theta_{2}=\theta_{0}+\Delta$, respectively. The empirical power $1-\hat{\beta }$ is estimated by the proportion of samples rejecting $H_{0}$ among the B=5000 simulation samples. Also, to check if the proposed Dunnett-type test accurately controls the FWER, we generate B=5000 samples of size *N* under $H_{0}$ (i.e., with Δ=0), and the empirical FWER $\hat{\alpha }$ is estimated by the proportion of samples rejecting $H_{0}$.

(Table 1 may be placed around here.)

The results of sample size calculations and simulations are summarized in Table 1. As expected, sample size increases in 1−β and decreases in |γ−1/2|, ρ, $\theta_{0}\left(>1/2\right)$, and $\Delta =\theta_{k}-\theta_{0}$ for k=1,2. From the simulation results, we find that our test statistic controls the FWER accurately and the calculated sample sizes are quite accurate in the sense that the empirical powers are close to the corresponding nominal power 1−β overall. For some design settings, however, the proposed test is slightly anti-conservative and the calculated sample size is slightly overpowered, especially if the calculated sample size is small and the allocation between cases and controls is very unbalanced (i.e., γ=0.1). The calculated sample sizes are never underpowered, at least for the design settings considered in this numerical study. Similar results were observed from numerical studies on two-sample version of this test [11]. In designing a study, slightly overpowered sample sizes are less of an issue than underpowered ones. If we want a more accurately powered sample size, we may calculate one using simulations. In this case, the sample size calculated from our formula can be used for validation of the sample size estimated from simulations.

**Example**: Suppose that there exists a biomarker A and we want to investigate whether we can improve its performance by adding a new biomarker B or C. So, biomarker 0 is A, 1 is A + B, and 2 is A+C. After a log-transformation and standardization with the mean of benign group and the common standard deviation as in Section 4, we assume that $\left(X_{0},X_{1},X_{2}\right)$ and $\left(Y_{0},Y_{1},Y_{2}\right)$ are independent trivariate normal random vectors with unit variances commonly, but with mean vectors (0,0,0) and $\left(\delta_{0},\delta_{1},\delta_{2}\right)$, respectively. More specifically, if $\left({\tilde{X}}_{0},{\tilde{X}}_{1},{\tilde{X}}_{2}\right)$ and $\left({\tilde{Y}}_{0},{\tilde{Y}}_{1},{\tilde{Y}}_{2}\right)$ denote the random vectors for the log-transformed biomarker data for malignant and benign groups, respectively, for k=0,1,2, let $\mu_{k}$ denote the mean of ${\tilde{Y}}_{k}$ and $s_{k}^{2}$ the common variance of ${\tilde{X}}_{k}$ and ${\tilde{Y}}_{k}$. Then, the standardized biomarker values will be $X_{k}=\left({\tilde{X}}_{k}-\mu_{k}\right)/s_{k}$ and $Y_{k}=\left({\tilde{Y}}_{k}-\mu_{k}\right)/s_{k}$. In the data analysis, $\mu_{k}$ and $s_{k}^{2}$ will be replaced by their sample estimates. Assuming that B and C add a similar amount of information to A, let $\mathrm{corr}\left(X_{0},X_{1}\right)=\mathrm{corr}\left(X_{0},X_{2}\right)=\mathrm{corr}\left(Y_{0},Y_{1}\right)=\mathrm{corr}\left(Y_{0},Y_{2}\right)=0.3$ and $\mathrm{corr}\left(X_{1},X_{2}\right)=\mathrm{corr}\left(Y_{1},Y_{2}\right)=0.3\sqrt{2}$. If we assume $\theta_{0}=0.7$ and $\theta_{1}=\theta_{2}=0.8$, the corresponding location-shift parameters are $\left(\delta_{0},\delta_{1},\delta_{2}\right)=\left(0.742,1.190,1.190\right)$. By assuming a prevalence of malignancy γ=0.5, we have $\rho_{12}=\mathrm{corr}\left({\hat{\theta }}_{1}-{\hat{\theta }}_{0},{\hat{\theta }}_{2}-{\hat{\theta }}_{0}\right)=0.65$. Using α=0.05, the required sample size for 80% of power is N=184, or m=γN=92 malignant cases and n=(1−γ)N=92 benign cases. From 5000 simulation samples, we observe an empirical FWER of $\hat{\alpha }=0.056$ under $H_{0}$ and power of $1-\hat{\beta }=0.888$ under $H_{a}$. We find that this sample size is somewhat overpowered.

## 6. Discussion

A Dunnett-type testing method is proposed to compare the performance of a control biomarker with K(≥2) new biomarkers using ROC curves. The test statistic is completely non-parametric and robust. Also proposed is a sample size calculation method for the Dunnett-type test. For a sample size calculation, we have to specify the distribution of the biomarkers. The biomarkers to be compared are collected from each subject, so they are usually correlated. Furthermore, the raw data of the biomarkers are usually positive, so their parameters, such as means, variances, and correlation coefficients, are complicatedly associated. As a result, it is difficult to specify the parameter values in a sample size calculation.

In our numerical studies, we assume equal correlation coefficients among the biomarkers for simplicity. But, the true correlation structure may be more complicated among real biomarkers. Our test statistic automatically accounts for any type of the true correlation structure by using the so-called GEE-type approach [14]. In sample size calculation, we have to specify the true correlation coefficients based on our best estimation. If we have pilot data, we may use the estimated correlation coefficients as the true ones in the sample size calculation. In this sense, we may consider a two-stage design of biomarker projects to estimate the correlation coefficients among the biomarkers from the first-stage data and calculate the sample size for the whole project combining the stage 1 and 2 data sets. In this case, we do not need a type I error adjustment for the final testing since we do not conduct any statistical testing at the first stage.

Since the AUC of ROC curves is a rank statistic, the AUCs and the proposed test statistic are invariant to a monotone transformation of biomarker data. Utilizing this nice property, Jung [11] proposed to calculate sample sizes based on the log-transformed data for the easy specification of the parameter values. We have extended his two-sample testing method to (K+1)-sample testing by controlling the FWER for multiple testing adjustment. Through simulations, we find that the Dunnett-type test controls the FWER accurately and the calculated sample sizes maintain the specified power closely overall. One-sided critical regions are used in the presentation of our method, but the extension to a two-sided test is straightforward.

To simplify the discussion, we have assumed that the transformed data follow a normal distribution, but it can be any distribution with a location-shift model between the benign and malignant groups. Whatever the transformed distribution, the correct distribution functions should be used in the sample size calculation. Note that there exists a transformation for any distribution transforming to a normal distribution, although it may not be a log-transformation. However, a log-transformation performs this goal relatively well in the real analysis of positive data.

The Dunnett test is popularly used in clinical trial or retrospective clinical studies to compare multiple experimental treatment groups with a common control group with a multiple testing adjustment. Usually, the groups to be compared are independent. For example, a Dunnett-type test and its sample size calculation method were proposed to compare K(≥2) experimental arms with a common control arm using a time-to-event endpoint [15]. In this paper, however, the K+1 biomarkers are to be compared since all the K+1 biomarkers are collected from each subject.

Contrary to the case of the Dunnett-type test, we may want to compare the K≥3 groups without a common control. In this case, we usually use a one-way ANOVA test. Jung and Hui [16] proposed a sample size calculation method for the ANOVA-type log-rank tests to compare K(≥3) treatment groups using a time-to-event endpoint.

Our Fortran program, developed for sample size calculation and simulations, is available from the author upon request.

## Figures and Tables

**Table 1:** Required sample size *N* (and empirical FWER level $\hat{\alpha }$ and power $1-\hat{\beta }$ from simulations) for 1-sided α=0.05 test of $H_{0}:\theta_{1}=\theta_{2}=\theta_{0}$ vs. $H_{a}:\left(\theta_{1}>\theta_{0}\right)\cup \left(\theta_{2}>\theta_{0}\right)$ given $r=\mathrm{corr}\left(X_{k},X_{k^{\prime }}\right)=\mathrm{corr}\left(Y_{k},Y_{k^{\prime }}\right)$ for $k\neq k^{\prime }$, AUCs $\left(\theta_{0},\theta_{1},\theta_{2}\right)$ under $H_{a}$, and prevalence of malignancy γ.

1−β	*r*	$\theta_{0}$	$\theta_{1}=\theta_{2}$	γ=0.1	γ=0.3	γ=0.5
0.8	0.2	0.6	0.7	703(0.057,0.806)	302(0.049,0.801)	253(0.052,0.804)
			0.75	299(0.063,0.813)	128(0.061,0.814)	108(0.056,0.812)
		0.7	0.8	565(0.059,0.823)	242(0.053,0.814)	204(0.048,0.814)
			0.85	234(0.065,0.841)	100(0.054,0.820)	84(0.058,0.829)
		0.8	0.9	370(0.063,0.847)	159(0.057,0.837)	134(0.053,0.848)
			0.95	153(0.070,0.917)	66(0.058,0.883)	55(0.051,0.879)
	0.4	0.6	0.7	540(0.052,0.813)	232(0.052,0.815)	195(0.051,0.797)
			0.75	231(0.058,0.823)	99(0.055,0.802)	84(0.049,0.807)
		0.7	0.8	440(0.055,0.830)	189(0.046,0.804)	159(0.055,0.816)
			0.85	186(0.065,0.846)	80(0.058,0.834)	67(0.061,0.821)
		0.8	0.9	300(0.060,0.863)	129(0.053,0.832)	108(0.052,0.845)
			0.95	135(0.070,0.929)	58(0.064,0.900)	49(0.049,0.898)
0.85	0.2	0.6	0.7	816(0.050,0.864)	350(0.059,0.856)	294(0.053,0.849)
			0.75	347(0.066,0.863)	149(0.051,0.858)	125(0.055,0.846)
		0.7	0.8	655(0.059,0.871)	281(0.053,0.866)	236(0.046,0.851)
			0.85	271(0.059,0.890)	117(0.057,0.862)	98(0.060,0.871)
		0.8	0.9	430(0.062,0.893)	184(0.060,0.881)	155(0.050,0.877)
			0.95	178(0.069,0.938)	77(0.055,0.933)	64(0.054,0.922)
	0.4	0.6	0.7	626(0.047,0.864)	269(0.056,0.858)	226(0.051,0.856)
			0.75	269(0.063,0.864)	115(0.055,0.863)	97(0.049,0.855)
		0.7	0.8	510(0.056,0.877)	219(0.056,0.854)	184(0.049,0.864)
			0.85	216(0.066,0.887)	93(0.057,0.877)	78(0.056,0.877)
		0.8	0.9	349(0.059,0.895)	150(0.054,0.886)	126(0.056,0.874)
			0.95	157(0.064,0.946)	67(0.051,0.939)	57(0.056,0.929)
0.9	0.2	0.6	0.7	969(0.055,0.912)	416(0.050,0.901)	349(0.049,0.903)
			0.75	412(0.058,0.905)	177(0.056,0.903)	149(0.053,0.909)
		0.7	0.8	779(0.055,0.906)	334(0.050,0.906)	281(0.049,0.905)
			0.85	322(0.060,0.922)	138(0.058,0.913)	116(0.058,0.909)
		0.8	0.9	511(0.052,0.932)	219(0.053,0.920)	184(0.048,0.919)
			0.95	212(0.065,0.971)	91(0.060,0.957)	76(0.051,0.959)
	0.4	0.6	0.7	744(0.056,0.904)	319(0.054,0.904)	268(0.054,0.895)
			0.75	319(0.061,0.905)	137(0.057,0.903)	115(0.055,0.908)
		0.7	0.8	607(0.057,0.911)	260(0.052,0.911)	219(0.048,0.913)
			0.85	257(0.054,0.924)	110(0.054,0.922)	93(0.057,0.918)
		0.8	0.9	415(0.062,0.937)	178(0.053,0.917)	150(0.049,0.927)
			0.95	186(0.066,0.974)	80(0.056,0.968)	67(0.048,0.965)

## Data Availability

No new data were created or analyzed in this study.
